# Dosimetric advantages for cardiac substructures in radiotherapy of esophageal cancer in deep-inspiration breath hold

**DOI:** 10.1007/s00066-024-02197-8

**Published:** 2024-02-05

**Authors:** Ahmed Allam Mohamed, Melina Nausikaa Douglas, Philipp Bruners, Michael J. Eble

**Affiliations:** 1 Department of Radiation Oncology, RWTH Aachen University Hospital, Pauwelstr. 30, 52074 Aachen, Germany; 2 Department of Diagnostic and Interventional Radiology, RWTH Aachen University Hospital, Aachen, Germany; 3 Center for Integrated Oncology Aachen, Bonn, Cologne and Duesseldorf (CIO ABCD), Aachen, Germany

**Keywords:** Cardiac toxicity, Neoadjuvant, CROSS protocol, Chemoradiotherapy, Pulmonary toxicity

## Abstract

**Background:**

Radiotherapy is one of the main treatment options for patients with esophageal cancer; however, it has been linked with an increased risk of cardiac toxicities. In the current study, we evaluated the effect of planning the radiation in deep-inspiration breath hold (DIBH) on the dose sparing of cardiac substructures and lung.

**Materials and methods:**

In this study, we analyzed 30 radiation therapy plans from 15 patients diagnosed with esophageal cancer planned for neoadjuvant radiotherapy. Radiation plans were generated for 41.4 Gy and delivered in 1.8 Gy per fraction for free-breathing (FB) and DIBH techniques. We then conducted a comparative dosimetric analysis, evaluating target volume coverage, the impact on cardiac substructures, and lung doses across the two planning techniques for each patient.

**Results:**

There was no significant disparity in target volume dose coverage between DIBH and FB plans. However, the D_mean_, D2%, and V30% of the heart experienced substantial reductions in DIBH relative to FB, with values of 6.21 versus 7.02 Gy (*p* = 0.011), 35.28 versus 35.84 Gy (*p* = 0.047), and 5% versus 5.8% (*p* = 0.048), respectively. The D_mean_ of the left ventricle was notably lower in DIBH compared to FB (4.27 vs. 5.12 Gy, *p* = 0.0018), accompanied by significant improvements in V10. Additionally, the D_mean_ and D2% of the left coronary artery, as well as the D2% of the right coronary artery, were significantly lower in DIBH. The dosimetric impact of DIBH on cardiac substructures proved more advantageous for middle esophageal (ME) than distal esophageal (DE) tumors.

**Conclusion:**

Radiotherapy in DIBH could provide a method to reduce the radiation dose to the left ventricle and coronaries, which could reduce the cardiac toxicity of the modality.

**Supplementary Information:**

The online version of this article (10.1007/s00066-024-02197-8) contains supplementary material, which is available to authorized users.

## Introduction

Esophageal cancer constitutes a significant clinical challenge, underscored by limited 5‑year survival rates that stand at 47% for localized cases, 26% for regional dissemination, and a stark 6% once metastasized [1]. Consequently, this malignancy ranks as the sixth leading cause of cancer-related deaths globally [2]. Recent decades have witnessed a histological shift in the incidence of esophageal cancer within affluent nations, transitioning predominantly from squamous cell carcinoma to adenocarcinoma [3].

The advent of integrated treatment strategies, in particular neoadjuvant radiochemotherapy, followed by surgical intervention has yielded significant improvements in survival rates for those with advanced esophageal cancer [4, 5]. However, radiation treatment of thoracic malignancies has been associated with an increased risk of cardiac diseases and cardiac mortality [6, 7]. Thoracic radiation therapy, particularly for esophageal cancer, has been correlated with a heightened incidence of cardiac toxicities and cardiac-related mortality. This correlation is evident when comparing patients who underwent radiation therapy to those who did not [8].

Indeed, multiple analyses have revealed an incidence of 16–25% for grade 3 or higher cardiac toxicity after radiation treatment in esophageal cancer, with the most common cardiac events being ischemic heart disease, coronary heart disease, pericardial effusion, and arrhythmia [9–11].

Emerging strategies are under exploration to mitigate cardiac toxicities associated with radiotherapy in esophageal cancer treatment. These include investigating proton therapy, which may reduce cardiac exposure [12], and considering radiotherapy exclusion in cases of adenocarcinoma histology [13]. Physiologically, deep inspiration leverages deformation and size reduction of the heart through negative intrathoracic pressure. Radiotherapy in deep-inspiration breath hold (DIBH) has already shown promising results in breast cancer treatment, where it has become a standard approach to protect the heart and the left anterior descending coronary artery (LAD) from radiation exposure in case of radiation treatment of left breast [14]. The same approach has been tested also for intrathoracic tumors, including lung cancer and thymic tumors [15, 16]. While previous studies have examined radiation in DIBH for esophageal cancer to reduce the mean dose to the heart and lung [17], a comprehensive dosimetric analysis of cardiac substructures during DIBH radiotherapy remains outstanding.

In the current study, we investigated the dosimetric advantage of radiotherapy for esophageal cancer in DIBH for heart substructures, namely the heart chambers and coronary arteries.

## Materials and methods

This analysis encompassed 30 planning computed tomographies (P-CTs) from 15 patients diagnosed with middle or lower esophageal cancer who were eligible to undergo P‑CT in free breathing (FB) and deep-inspiration breath hold (DIBH) between January 2021 and December 2022. Initially, FB P‑CTs were performed on a 16-slice CT scanner (Brilliance CT Big Bore Oncology, Philips Medical Systems, Inc., Cleveland, Ohio, USA) employing parameters of 120 kV, 146 mAs, a pitch of 0.813, and a slice thickness of 3 mm, following the administration of iodinated contrast media. Subsequently, the DIBH P‑CTs were conducted in the same session using an optical surface imaging system, Catalyst™ (CRAD, Uppsala, Sweden), to monitor sternal motion during DIBH CT acquisition. Planning and diagnostic CTs along with available positron-emission tomography (PET) scans were anonymized and imported into the Pinnacle3 treatment planning system (V.14.0; Philips Healthcare, Amsterdam, Netherlands). The analysis was approved by the local ethics committee (Faculty of Medicine, RWTH Aachen University, approval sign: EK 23-088).

### Volume delineations

A radiation oncologist (AM) generated all target volume (TV) delineations to ensure uniformity, and a second radiation oncologist (ME) reviewed them. The gross tumor volume (GTV) included contrast-enhanced thickening and/or FDG-avid lesions on the esophageal wall (GTVp) and enlarged regional lymph nodes greater than 1 cm in short axis diameter and/or FDG avid (GTVn), visualized using a thoracic mediastinal window (window width: 401 HU; window level: 800 HU). Clinical target volumes (CTV) were delineated by extending GTVp by 4 cm craniocaudally and 1.5 cm radially (CTVp) and GTVn by 1.5 cm in all directions (CTVn), excluding the lungs, heart, aorta, and trachea if uninvolved. The planning target volume (PTV) was defined by further expanding the combined CTV (CTVp+n) by 5 mm in all directions.

Subsequently, lung and heart substructures, including the left and right ventricles (LV and RV, respectively), left and right atria (LA and RA, respectively), right coronary (RCA), left coronary (LCA), left anterior descending (LAD), and left circumflex coronary (LCX) arteries were contoured using contouring guidelines as previously described [18] and revised by a radiologist (PB).

### Treatment planning

Thirty volumetric modulated arc therapy (VMAT) plans were created. Each patient had one plan based on FB-CT and another on DIBH-CT using the collapsed cone algorithm with a 3 × 3 × 3 mm^3^ dose grid, all by the same medical physicist (MD). Each plan consisted of two full coplanar arcs at a photon energy of 6 MV, with a normalization mode of 100% and a prescribed PTV dose of 41.4 Gy over 23 fractions. The optimization aimed for uniform PTV coverage with the prescribed dose, ensuring 95% of the dose encompassed 100% of the CTV and over 95% of the PTV.

The following dose–volume parameters were used for plan evaluation:

- D2%: the near maximum dose.
- D98%: the near minimum dose.
- D_mean_: the mean dose of TV/OAR.
- Dx: the dose to defined (x) volume from TV/OAR.
- Vx_Gy_: the volume of TV/OAR that receives a defined (x) dose.

Dose constraints for organ at risk (OAR) were spinal canal D2%< 45 Gy, liver D_mean_ < 32 Gy, lung D_mean_ < 17.5 and D20% < 20 Gy, and heart D_mean_ < 26 Gy.

### Dosimetric analysis

A dose–volume histogram (DVH) was generated for each plan. Different dosimetric/volumetric data were extracted from DVHs and used for plan evaluation, including

- PTV volume, PTV D2% and D98% median dose (D50%), and the volume of PTV enclosed by reference isodose line (V95%).
- the conformity index RTOG (CI) = VRI/TV, where VRI is the reference isodose volume (95% isodose volume) and TV is the target volume (PTV).
- the homogeneity index RTOG (HI) = Imax/RI, where Imax is the maximum isodose in the target and RI is the reference isodose.

In addition, dosimetric data from the cardiac substructure and lungs were extracted for comparison between plans in FB and DIBH for each patient, which included the following:

- the entire heart: volume, D_mean_, V30 Gy, and D2%.
- the heart chambers (ventricles and atria): D_mean_, D2%, and V10,20,30, and 40 Gy
- coronary arteries: D_mean_, D2%, and V5,10,20,30, and 40 Gy.
- lung: volume, D_mean_, and V20 Gy.

### Statistical analysis

All collected data were transferred to Microsoft Excel (Microsoft Corporation, Redmond, WA, U.S.) for subsequent analysis. We present the data as means with their respective standard deviations (± SD). To assess the distribution of the data, the Shapiro–Wilk test was conducted. Data with a *p*-value greater than 0.05 were deemed to follow a normal distribution (parametric data), whereas those with a *p*-value less than 0.05 were classified as non-parametric. For parametric datasets, a paired *t*-test was used to compare the means of each parameter. Conversely, the Wilcoxon signed-rank test was employed for datasets where at least one group of compared data was non-parametric.

## Results

Thirty radiation plans from 15 patients were evaluated in the current analysis: 15 in DIBH and 15 in FB. All participants met the criteria for neoadjuvant radiotherapy (either T3 tumor and/or node positive) including 9 patients with squamous cell histology and 6 patients with adenocarcinoma histology (Table 1). Twelve patients underwent PET scans for radiation planning within 1 week of P‑CT, which were used to assist in target volume delineation.

**Table 1:** Selected patient characteristics

Patient number	Age (years)	Histology	Stage	Location	Distance from the incisor teeth (cm)
#1	66	Adenocarcinoma	T3N1	Middle	26
#2	61	Squamous cell cancer	T3N2	Middle	27
#3	77	Squamous cell cancer	T3N0	Middle	30
#4	82	Squamous cell cancer	T3N0	Middle	30
#5	79	Squamous cell cancer	T3N1	Middle	26
#6	79	Squamous cell cancer	T3N0	Middle	25
#7	83	Squamous cell cancer	T3N2	Middle	29
#8	73	Squamous cell cancer	T3N1	Distal	32
#9	70	Squamous cell cancer	T3N0	Middle	25
#10	76	Adenocarcinoma	T3N0	Distal	39
#11	73	Squamous cell cancer	T3N2	Middle	25
#12	79	Squamous cell cancer	T3N2	Middle	25
#13	86	Adenocarcinoma	T3N0	Distal	34
#14	53	Adenocarcinoma	T3N2	Middle	28
#15	88	Adenocarcinoma	T3N0	Distal	34

The average volume of PTV in DIBH was 342.09 cm^3^ (SD ± 144.12), while for FB it was 363.73 cm^3^ (SD ± 132.76), with a *p*-value of 0.088. Dosimetric parameters assessing target volume coverage—including D2%, D98%, D50%, and V95%—demonstrated comparable results between DIBH and FB plans (Table 2). The mean HI was consistent at 1.1 for both cohorts (*p*-value: 0.125). Additionally, the average CI was 1.02 for plans in DIBH and 1.03 for plans in FB (*p*-value: 0.33), suggesting similar conformity. The average monitor units per plan showed no significant variance, with 507.7 for DIBH and 535.23 for FB (*p*-value: 0.107).

**Table 2:** Characteristics of treatment plans between deep-inspiration breath hold (DIBH) and free breathing (FB)

Volumetric/dosimetric parameter	Mean in DIBH (range)	Mean in FB (range)	*P*-value
PTV	342.09 (175–626) cm^3^	363.73 (179–597) cm^3^	0.088^a^
D2%	42.78 (42.4–43.1) Gy	42.69 (42.3–43.4) Gy	0.81^a^
D98%	38.85 (38.4–39.4) Gy	38.94 (38.2–39.5) Gy	0.35^a^
D50%	41.42 (41.2–41.6) Gy	41.43 (41.3–41.6) Gy	0.325^a^
V95%	96.27 (95–99)%	96.53 (95–98)%	0.16^a^
CI	1.02 (0.88–1.26)	1.03 (0.92–1.1)	0.33^a^
HI	1.1 (1.09–1.11)	1.1 (1.09–1.11)	0.125^a^
MU	507.71 (434.1–660.8)	535.23 (438.1–639.7)	0.107^b^

*CI* conformity index, *Hi* homogeneity index, *MU* monitor unit, *D2%* near maximum dose, *D98%* near minimum dose, *D50%* dose to 50% volume from target volume, *V95%* volume of PTV that receives 95% dose

^a^paired t‑test

^b^Wilcoxon signed-rank test

### Dosimetric outcomes for OAR

**Fig. 1:**
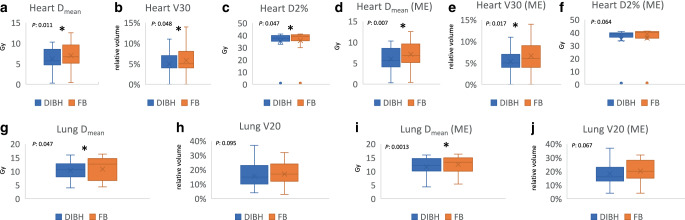
Boxplot showing the heart D_mean_ (**a**), V30 (**b**), and D2% (**c**) irrespective of tumor location; heart D_mean_ (**d**), V30 (**e**), and D2% (**f**) for middle esophageal (ME) tumors; and lung D_mean_ (**g**, **i**) and V20 (**h**, **j**) irrespective of tumor location and for middle esophageal (ME) tumor, respectively, between the deep-inspiration breath hold plans (DIBH) in *blue* and free breathing plans (FB) in *orange*, *asterisk* *P*-value < 0.05

The mean cardiac volume was significantly reduced in DIBH compared to FB, with values of 831.5 cm^3^ and 885.4 cm^3^, respectively (*p*-value: 0.012). Similarly, the average mean dose to the heart (heart D_mean_) was significantly lower in DIBH-plans, at 6.21 Gy, compared to 7.02 Gy in FB-plans (*p*-value: 0.011). Moreover, V30 Gy and D2% of the heart in DIBH (5% and 35.28 Gy) were significantly lower than FB (5.8% and 35.84 Gy), with *p*-values 0.048 and 0.047, respectively (Fig. 1, Table 3).

**Table 3:** Relevant dosimetric outcomes of planning in deep-inspiration breath hold (DIBH) and free breathing (FB) for the whole cohort, middle esophageal (ME), and distal esophageal (DE) tumors

	DIBH	FB	*P*-value
**Entire cohort**			
*Whole heart*			
Size	831.5 cm^3^	885.4 cm^3^	0.012^b^*
D_mean_	6.21 Gy	7.02 Gy	0.011^a^*
V30 Gy	5%	5.8%	0.048^a^*
D2%	35.28 Gy	35.84 Gy	0.047^b^*
*Right ventricle*			
D_mean_	2.65 Gy	3.7 Gy	0.2^b^
*Left ventricle*			
D_mean_	4.27 Gy	5.12 Gy	0.0018^a^*
V10 Gy	8.5%	11.5%	0.011^a^*
V20 Gy	2.6%	4%	0.079^b^
*Left coronary artery*			
D_mean_	3 Gy	3.4 Gy	0.019^a^*
V5 Gy	0.3%	10.8%	0.1^b^
D2%	3.4 Gy	5.5 Gy	0.021^b^*
*Right coronary artery*			
D_mean_	2.23 Gy	2.99 Gy	0.064^b^
D2%	3.86 Gy	4.96 Gy	0.03^b^*
*Lung*			
Volume	5138 cm^3^	3605 cm^3^	<0.00001^a^*
D_mean_	10.3	10.78	0.047^a^*
V20 Gy	15.6%	16.9%	0.09^a^
**Middle esophageal (ME) tumors**			
*Whole heart*			
Size	715.6 cm^3^	738.9 cm^3^	0.09^a^
D_mean_	6.02 Gy	7.13 Gy	0.007^a^*
V30 Gy	5.3%	6.6%	0.0177^a^*
D2%	34.99	35.82	0.064^a^
*Left ventricle*			
D_mean_	3.79 Gy	4.85 Gy	0.0018^a^*
V10 Gy	6.8%	11%	0.014^b^*
V20 Gy	2%	4%	0.035^b^*
D2%	14.19 Gy	16.82 Gy	0.041^b^*
*Right atrium*			
D_mean_	5.73 Gy	7.11 Gy	0.087^a^
D2%	18.6 Gy	24.02 Gy	0.019^a^*
*Left coronary artery*			
D_mean_	3.14 Gy	3.54 Gy	0.016^b^*
D2%	3.55 Gy	5.88 Gy	0.067^b^
*Left circumflex coronary*			
D_mean_	11.4 Gy	13.1 Gy	0.09^a^
V20 Gy	21%	28%	0.025^a^*
*Right coronary artery*			
D_mean_	1.64 Gy	2.11 Gy	0.057^a^
D2%	2.15 Gy	3.03 Gy	0.046^a^*
*Lung*			
Volume	5184	3913	< 0.00001^a^*
D_mean_	11.63 Gy	12.53 Gy	0.001^a^*
V20 Gy	18.2%	20.2%	0.067^a^
**Distal esophageal (DE) tumors**			
*Left anterior descending artery*			
D2%	2.52 Gy	2.88 Gy	0.015^a^*
*Left circumflex coronary*			
V20 Gy	50%	29.7%	0.059^a^
V30 Gy	37%	14.5%	0.059^a^

^a^paired *t*-test

^b^Wilcoxon signed-rank test

*p-value < 0.05

**Fig. 2:**
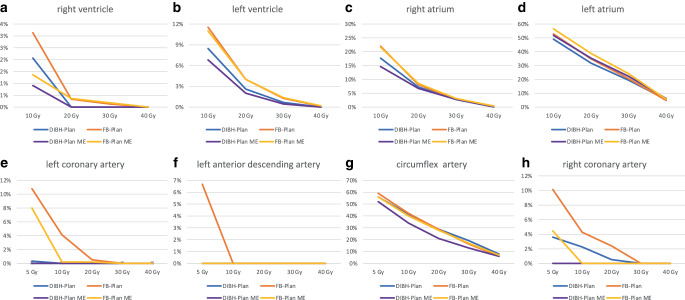
Linear diagrams representing relative volume in relation to dose for the right ventricle (**a**), left ventricle (**b**), right atrium (**c**), left atrium (**d**), left coronary (**e**), left anterior descending (**f**), left circumflex (**g**), and right coronary artery (**h**) in deep-inspiration breath hold (*DIBH*) and free breathing (*FB*) for both the entire cohort and middle third esophageal tumor (*ME*)

Regarding the dosimetric impact on cardiac chambers, DIBH plans demonstrated a significantly lower D_mean_ to the LV compared to FB plans, with a mean dose of 4.27 Gy versus 5.12 Gy, respectively (*p*-value: 0.0018). Also, V10 and V20 Gy of LV were reduced in DIBH, with 8.5% compared to 11.5% and 2.6% versus 4% in FB, with *p*-values of 0.011 and 0.079, respectively. However, for the right ventricle and both atria, no significant differences in dose–volume metrics were observed between the DIBH and FB plans (Fig. 2, Table 3).

The D_mean_ and D2% of the LCA were significantly lower in DIBH at 3 Gy and 3.4 Gy compared to FB at 3.4 Gy and 5.5 Gy, with *p*-values of 0.019 and 0.021, respectively. Additionally, planning in DIBH yielded lower D_mean_ and D2% for the RCA at 2.23 Gy and 3.86 Gy, as opposed to 2.99 Gy and 4.96 Gy in FB, with *p*-values of 0.064 and 0.03, respectively (Table 3). The dosimetric parameters for the other coronary arteries did not demonstrate any significant differences between the FB and DIBH plans (Fig. 2, Table 3).

Further, the mean lung volume was higher for DIBH compared to FB (5138 vs. 3605 cm^3^, *p*-value < 0.00001), and this was linked to a significantly reduced D_mean_ in DIBH vs. FB, 10.3 vs. 10.8 Gy (*p*-value: 0.047; Fig. 1, Table 3).

Next, we analyzed the dosimetric parameters based on tumor location, where the tumor location was categorized into middle esophageal (ME) or distal esophageal (DE) tumors (proximal edge of the tumor < 32 vs. > 32 cm from the incisor teeth, respectively). When planning in DIBH versus FB for ME tumors, we observed a significant improvement in heart D_mean_ (6.02 vs. 7.13 Gy, *p*-value 0.007) and heart V30 Gy (5.3% vs. 6.6%, *p*-value 0.017), along with a reduction in heart D2% (34.99 Gy vs. 35.8 Gy, *p*-value 0.064). Moreover, the LV demonstrated significantly lower D_mean_, D2%, V10, and V20 Gy in DIBH (3.79 Gy, 14.19 Gy, 6.8%, and 2%, respectively) compared to FB (4.85 Gy, 16.82 Gy, 11%, and 4%, respectively), with respective *p*-values of 0.0018, 0.041, 0.014, and 0.035. The RA exhibited a reduced D_mean_ and D2% in DIBH (5.73 Gy and 18.6 Gy) compared to FB (7.11 Gy and 24.02 Gy), with *p*-values of 0.087 and 0.019. For ME tumors, DIBH planning yielded lower D_mean_ and D2% for the LCA (3.14 vs. 3.54 Gy and 3.55 vs. 5.88 Gy, *p*-values 0.016 and 0.067, respectively), lower D_mean_ and V20 for the LCX (11.4 vs. 13.1 Gy, 21% vs. 28%, *p*-values 0.09 and 0.025 respectively), and decreased D_mean_ and D2% for the RCA (1.6 vs. 2.1 Gy, 2.15 vs. 3.03 Gy, *p*-values 0.057 and 0.046, respectively).

Nonetheless, the D_mean_ and V20 Gy of the lung in DIBH were 11.6 Gy and 18.2%, and in FB they were 12.5 Gy and 20.2%, with *p*-values of 0.0013 and 0.067, respectively.

Conversely, planning in DIBH for DE tumors only had a dosimetric advantage with regard to D2% of the LAD (2.52 Gy vs. 2.88, *p*-value 0.015) and a trend for improved V20 and V30 of LCX in FB compared to DIBH (50% vs. 29.7% and 37% vs. 14.5%, *p*-value: 0.059 and 0.059, respectively). All other cardiac, subcardiac, or pulmonary parameters evidenced comparable outcomes for DIBH and FB.

## Discussion

Cardiac toxicity after radiation therapy in esophageal cancer has emerged as a significant concern [9]. In their analysis, Cai et al. revealed compelling evidence demonstrating that the radiation doses of LV, LAD, and LCX are strong predictors of severe cardiac events of grade 3 or higher [19]. Nonetheless, Burke et al. conducted a prospective study to delve into the structural cardiac alterations using serial cardiac MRI for patients with esophageal cancer treated with neoadjuvant chemoradiotherapy [20]. They found fibrotic and ischemic myocardial changes in almost one third of the patients; these changes were significantly associated with an increase in left ventricular end-systolic volume (LVESV).

The current study aimed to evaluate the possible dosimetric advantage of planning DIBH for cardiac substructures in patients undergoing neoadjuvant radiotherapy for locally advanced esophageal cancer. A previous study assessed the dosimetric advantage of DIBH for OARs in esophageal cancer radiotherapy, concluding a correlation with some dosimetric benefits such as a lower heart V40 compared to planning in FB [17]. However, detailed analysis concerning the subcardiac structures remains scant in the literature.

**Fig. 3:**
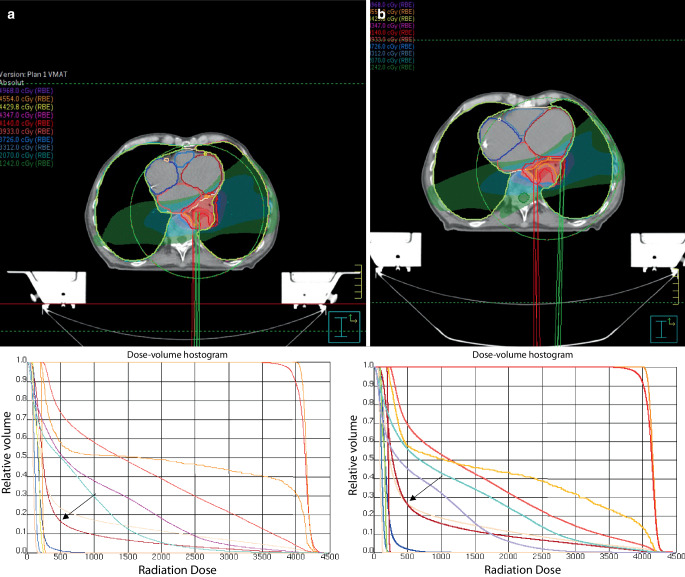
Dose color wash from example of VMAT plans for a middle third esophageal tumor in free breathing (FB) (**b**) and deep-inspiration breath hold (DIBH) (**a**) as well as the dose–volume histograms (DVH) from both plans (PTV in *red*, CTV in *orange*, heart in seashell, left ventricle in *maroon red*, left atrium in *tomato red*, right atrium in *blue*, right ventricle in *steel blue*, left coronary in *dark red*, left anterior descending artery in *yellow-green*, circumflex artery in *light orange*, right coronary in *light pink*, right lung in *lavender*, and left lung in *sky blue*). The increased lung volumes and negative intrathoracic pressure result in a reduction of the heart size with deformation, which resulted in a reduction of the beam path through the left ventricle (*arrow* on DVHs)

The current analysis confirmed that planning in DIBH provides superior cardiac sparing compared to FB. The mean heart volume, D_mean_, V30 Gy, and D2% of the entire heart were significantly lower in DIBH versus FB. This could be explained by the reduction in intrathoracic pressure and atrial filling associated with increased lung volumes, leading to heart deformation and a consequent decrease in heart volume, thereby diminishing cardiac radiation exposure, as demonstrated in Fig. 3 [21]. These results align with other studies analyzing mediastinal or lung tumors [16, 22]. The clinical relevance of these consistent differences in radiation exposure to the heart between planning in DIBH and FB, even when the magnitude is small, should be considered in light of the results of the analysis from Darby et al., where major coronary events increased linearly by 7.4% per increment with 1 Gy of heart D_mean_ in female patients with breast cancer who received adjuvant radiation [23].

In examining subcardiac structures, DIBH demonstrated significant reductions in both D_mean_ and D2% for the LCA, as well as in D2% for the RCA, when compared to FB. Furthermore, D_mean_ and V10 of LV were significantly lower in DIBH relative to FB. These results gain particular significance in the context of the findings of Cai et al. [19], where they showed LV to be a critical organ for radiation-induced cardiac toxicity. Radiation treatment in DIBH could significantly reduce the dose to LV, and that would reflect on the incidence of cardiac toxicity.

Remarkably, dosimetric analysis revealed that planning in DIBH for ME tumors conferred greater benefits to cardiac substructures than DE tumors. Not only were the D_mean_ and V30 Gy of the entire heart reduced, but also the D_mean_, D2%, V10, and V20 Gy of the LV, along with the D2% of the RA, showed significantly lower doses in DIBH. Furthermore, the D_mean_ of the LCA, V20 of the LCX, and D2% of the RCA were lower in the DIBH group.

The lung volume increased during DIBH, resulting in a significantly lower D_mean_ to the lungs across the entire cohort, particularly in patients with middle-third esophageal tumors.

In contrast, while planning in DIBH for DE tumors revealed a dosimetric improvement for D2% of the LAD, there was a trend toward higher doses observed for V20 and V30 of the LCX during DIBH.

## Conclusion

This study substantiates that the DIBH technique facilitates significant cardiac sparing during neoadjuvant radiotherapy for patients with locally advanced esophageal cancer. It underscores the necessity of tumor position within the esophagus as a determinant for the efficacy of DIBH in mitigating cardiac and pulmonary doses. Future prospective studies are warranted to corroborate these observations and refine the application of DIBH in the radiotherapeutic management of esophageal cancer.

### Supplementary Information

Supplementary Table 1: Dosimetric outcomes of planning in deep inspiration breath hold  (DIBH) and free breathing (FB) for the patients for the entire cohort. Supplementary Table 2: Dosimetric outcomes of planning in deep inspiration breath hold  (DIBH) and free breathing (FB) for the patients with middle esophageal tumors.

## References

[1] Survival Rates for Esophageal Cancer | Esophageal Cancer Outlook. https://www.cancer.org/cancer/esophagus-cancer/detection-diagnosis-staging/survival-rates.html#references. Accessed 12 Mar 2023

[2] Bray F, Ferlay J, Soerjomataram I, Siegel RL, Torre LA, Jemal A (2018). Global cancer statistics 2018: GLOBOCAN estimates of incidence and mortality worldwide for 36 cancers in 185 countries. CA Cancer J Clin.

[3] Lin Y, Wang H, Fang K, Zheng Y, Wu J (2022). International trends in esophageal cancer incidence rates by histological subtype (1990–2012) and prediction of the rates to 2030. Esophagus.

[4] van Hagen P, Hulshof MCCM, van Lanschot JJB, Steyerberg EW, van Berge Henegouwen MI, Wijnhoven BPL, Richel DJ, Nieuwenhuijzen GAP, Hospers GAP, Bonenkamp JJ (2012). Preoperative Chemoradiotherapy for Esophageal or Junctional Cancer. N Engl J Med.

[5] Reynolds JV, Preston SR, O’Neill B, Lowery MA, Baeksgaard L, Crosby T, Cunningham M, Cuffe S, Griffiths GO, Roy R (2021). Neo-AEGIS (Neoadjuvant trial in Adenocarcinoma of the Esophagus and Esophago-Gastric Junction International Study): Preliminary results of phase III RCT of CROSS versus perioperative chemotherapy (Modified MAGIC or FLOT protocol). (NCT01726452). J Clin Oncol.

[6] Piroth MD, Baumann R, Budach W, Dunst J, Feyer P, Fietkau R, Haase W, Harms W, Hehr T, Krug D (2019). Heart toxicity from breast cancer radiotherapy. Strahlenther Onkol.

[7] Jaworski C, Mariani JA, Wheeler G (2013). Cardiac complications of thoracic irradiation. J Am Coll Cardiol.

[8] Gharzai L, Verma V, Denniston KA, Bhirud AR, Bennion NR, Lin C (2016). Radiation therapy and cardiac death in long-term survivors of esophageal cancer: an analysis of the surveillance, epidemiology, and end result database. Bruns H, editor. Plos One.

[9] Wang X, Palaskas NL, Yusuf SW, Abe J-I, Lopez-Mattei J, Banchs J, Gladish GW, Lee P, Liao Z, Deswal A (2020). Incidence and onset of severe cardiac events after radiotherapy for esophageal cancer. J Thorac Oncol.

[10] Hayashi Y, Iijima H, Isohashi F, Tsujii Y, Fujinaga T, Nagai K, Yoshii S, Sakatani A, Hiyama S, Shinzaki S (2019). The heart’s exposure to radiation increases the risk of cardiac toxicity after chemoradiotherapy for superficial esophageal cancer: A retrospective cohort study. BMC Cancer.

[11] Witt JS, Jagodinsky JC, Liu Y, Yadav P, Kuczmarska-Haas A, Yu M, Maloney JD, Ritter MA, Bassetti MF, Baschnagel AM (2019). Cardiac toxicity in operable esophageal cancer patients treated with or without chemoradiation. Am J Clin Oncol.

[12] Lin SH, Hobbs BP, Verma V, Tidwell RS, Smith GL, Lei X, Corsini EM, Mok I, Wei X, Yao L (2020). Randomized phase IIB trial of proton beam therapy versus intensity-modulated radiation therapy for locally advanced esophageal cancer. J Clin Oncol.

[13] Hoeppner J, Lordick F, Brunner T, Glatz T, Bronsert P, Röthling N, Schmoor C, Lorenz D, Ell C, Hopt UT et al (2016) ESOPEC: Prospective randomized controlled multicenter phase III trial comparing perioperative chemotherapy (FLOT protocol) to neoadjuvant chemoradiation (CROSS protocol) in patients with adenocarcinoma of the esophagus (NCT02509286). BMC Cancer 16(1) 10.1186/s12885-016-2564-y PMC4952147 27435280

[14] Wolf J, Stoller S, Lübke J, Rothe T, Serpa M, Scholber J, Zamboglou C, Gkika E, Baltas D, Juhasz-Böss I (2022). Deep inspiration breath-hold radiation therapy in left-sided breast cancer patients: a single-institution retrospective dosimetric analysis of organs at risk doses. Strahlenther Onkol.

[15] Josipovic M, Aznar MC, Thomsen JB, Scherman J, Damkjaer SMS, Nygård L, Specht L, Pøhl M, Persson GF (2019) Deep inspiration breath hold in locally advanced lung cancer radiotherapy: validation of intrafractional geometric uncertainties in the INHALE trial. Br J Radiol 92(1104) 10.1259/bjr.20190569 PMC6913352 31544478

[16] Yan D, Ning L, Chen Y, Ke S, Huang H, Wang L, Yan S (2022). Analysis of deep inspiration breath-hold technique to improve dosimetric and clinical advantages in postoperative intensity-modulated radiation therapy for thymomas. Quant Imaging Med Surg.

[17] Lorchel F, Dumas JL, Noë A, Wolf D, Bosset JF, Aletti P (2006). Dosimetric consequences of breath-hold respiration in conformal radiotherapy of esophageal cancer. Phys Med.

[18] Duane F, Aznar MC, Bartlett F, Cutter DJ, Darby SC, Jagsi R, Lorenzen EL, Mcardle O, Mcgale P, Myerson S (2017). Cardiac contouring atlas A cardiac contouring atlas for radiotherapy. Radiother Oncol.

[19] Cai G, Li C, Li J, Yang J, Li C, Sun L, Li J, Yu J, Meng X (2023). Cardiac substructures dosimetric predictors for cardiac toxicity after definitive radiotherapy in esophageal cancer. Int J Radiat Oncol.

[20] Burke AM, Yeh C, Kim S, Bergquist P, Krishnan P, Barac A, Srichai MB, Unger K (2020). A prospective study of early radiation associated cardiac toxicity following neoadjuvant chemoradiation for distal esophageal cancer. Front Oncol.

[21] Carroll RG (2007). Integrated cardiovascular function. Elsevier’s Integr Physiol.

[22] Persson GF, Scherman Rydhög J, Josipovic M, Maraldo MV, Nygård L, Costa J, Berthelsen AK, Specht L, Aznar MC (2016). Deep inspiration breath-hold volumetric modulated arc radiotherapy decreases dose to mediastinal structures in locally advanced lung cancer. Acta Oncol.

[23] Darby SC, Ewertz M, McGale P, Bennet AM, Blom-Goldman U, Brønnum D, Correa C, Cutter D, Gagliardi G, Gigante B (2013). Risk of is che mic heart disease in women after radiotherapy for breast cancer (abstract from the clinical trial service unit mark; the department of medical Epide-miology and biostatistics). N Engl J Med.

