# Supplementary material for: Dosimetric advantages for cardiac substructures in radiotherapy of esophageal cancer in deep-inspiration breath hold
Source: Strahlenther Onkol. 2024 Feb 5;200(7):624–32. doi: 10.1007/s00066-024-02197-8 (PMC11186874; doi:10.1007/s00066-024-02197-8)
Supplement: Supplementary file 1 — Supplementary Table 1: Dosimetric outcomes of planning in deep inspiration breath hold (DIBH) and free breathing (FB) for the patients for the entire cohort. Supplementary Table 2: Dosimetric outcomes of planning in deep inspiration breath hold (DIBH) and free breathing (FB) for the patients with middle esophageal tumors. [file 66_2024_2197_MOESM1_ESM.docx]

**Supplementary material**

|  | **DIBH-P** | **FB-P** | **P Value** |
| --- | --- | --- | --- |
| **Whole Heart** |  |  |  |
| Size | 831.5 cm^3^ | 885.4 cm^3^ | 0.012 ^§, *^ |
| D_mean_ | 6.21 Gy | 7.02 Gy | 0.0114 ^+, *^ |
| V30 Gy | 2.5% | 3.4% | 0.048 ^+,*^ |
| D2% | 35.28 Gy | 35.84 Gy | 0.047 ^§, *^ |
| **Right ventricle** |  |  |  |
| D_mean_ | 2.65 Gy | 3.7 Gy | 0.2 ^§^ |
| V10 Gy | 2% | 3.1% | 0.41 ^§^ |
| V20 Gy | 0 | 0.3% | 0.11 |
| V30 Gy | 0 | 0.1% | 0.16 |
| D2% | 4.4 Gy | 6.8 Gy | 0.25 ^§^ |
| **Left ventricle** |  |  |  |
| D_mean_ | 4.27 Gy | 5.12 Gy | 0.0018 ^+,*^ |
| V10 Gy | 8.5% | 11.5% | 0.011 ^+,*^ |
| V20 Gy | 2.6% | 4% | 0.079 ^§^ |
| V30 Gy | 0.6% | 1.3% | 0.17 ^§^ |
| V40 Gy | 0 | 0.1% | 0.34 ^§^ |
| D2% | 17.16 Gy | 18.61 Gy | 0.16 ^+^ |
| **Right atrium** |  |  |  |
| D_mean_ | 6.38 Gy | 7.1 Gy | 0.2 ^+^ |
| V10 Gy | 17.6% | 22% | 0.18 ^+^ |
| V20 Gy | 7.2% | 8.1% | 0.78 ^§^ |
| V30 Gy | 2.7% | 2.8% | 0.9 ^§^ |
| V40 Gy | 0.06% | 0.4% | 0.17^§^ |
| D2% | 22.25 | 24.75 | 0.16^§^ |
| **Left atrium** |  |  |  |
| D_mean_ | 15.08 Gy | 15.94 Gy | 0.84^§^ |
| V10 Gy | 49% | 52.8% | 0.62^§^ |
| V20 Gy | 31.7% | 34.8% | 0.2 ^+^ |
| V30 Gy | 19.4% | 20.5% | 0.34^+^ |
| V40 Gy | 5.4% | 4.8% | 0.25 ^+^ |
| D2% | 37.9 Gy | 37.34 Gy | 0.4 ^§^ |
| **Left coronary** |  |  |  |
| D_mean_ | 3 Gy | 3.4 Gy | 0.019 ^+,*^ |
| V5 Gy | 0.3% | 10.8% | 0.1 ^§^ |
| V10 Gy | 0 | 1.2% | 0.37 ^§^ |
| V20 Gy | 0 | 0.13% | 1 ^§^ |
| D2% | 3.4 Gy | 5.5 Gy | 0.021 ^§,*^ |
| **Left anterior descending** |  |  |  |
| D_mean_ | 1.8 Gy | 1.85 Gy | 0.22 ^+^ |
| D2% | 2.81 Gy | 2.93 Gy | 0.13 ^+^ |
| **Circumflex artery** |  |  |  |
| D_mean_ | 13.85 Gy | 13.37 Gy | 0.37 ^+^ |
| V5 Gy | 56% | 58.9% | 0.5 ^§^ |
| V10 Gy | 41.2% | 42.2% | 0.4 ^+^ |
| V20 Gy | 28% | 28% | 0.48 ^§^ |
| V30 Gy | 19.2% | 16.3% | 0.84 ^+^ |
| V40 Gy | 7.9% | 6.2% | 0.9 ^+^ |
| D2% | 31.28 Gy | 31.7 GY | 0.76 ^+^ |
| **Right coronary** |  |  |  |
| D_mean_ | 2.23 Gy | 2.99 Gy | 0.064 ^§^ |
| V5 Gy | 3.6% | 10% | 0.42 ^§^ |
| D2% | 3.86 Gy | 4.96 Gy | 0.03 ^§,*^ |
| **Lung** |  |  |  |
| Volume | 5138 cm^3^ | 3605 cm^3^ | < 0.00001 ^+,*^ |
| D_mean_ | 10.3 | 10.78 | 0.047 ^+,*^ |
| V20 Gy | 15.6% | 16.9% | 0.09 ^+^ |

**Supp. Table 1**: Dosimetric outcomes of planning in deep inspiration breath hold (DIBH) and free breathing (FB) for the patients with middle and distal esophageal tumors, ^+^ paired t test, ^§^ Wilcoxon signed-rank test, * : p value < 0.05

|  | **DIBH-P** | **FB-P** | **P Value** |
| --- | --- | --- | --- |
| **Whole Heart** |  |  |  |
| Size | 715.6 cm^3^ | 738.9 cm^3^ | 0.09 ^+^ |
| D_mean_ | 6.02 Gy | 7.13 Gy | 0.007 ^+,*^ |
| V30 Gy | 5.3% | 6.6% | 0.017 ^+,*^ |
| D2% | 34.99 Gy | 35.8 | 0.064 ^+^ |
| **Right ventricle** |  |  |  |
| D_mean_ | 2.16 Gy | 3.5 Gy | 0.22^§^ |
| V10 Gy | 0.9% | 1.3% | 0.78^§^ |
| V20 Gy | 0 | 0..3% | 0.17^§^ |
| V30 Gy | 0 | 0.18% | 0.17^§^ |
| D2% | 3.88 Gy | 6.7 Gy | 0.2^§^ |
| **Left ventricle** |  |  |  |
| D_mean_ | 3.79 Gy | 4.85 Gy | 0.0018 ^+,*^ |
| V10 Gy | 6.8 % | 11 % | 0.014 ^§,*^ |
| V20 Gy | 2% | 4% | 0.035 ^§,*^ |
| V30 Gy | 0.45% | 1.3% | 0.18 ^§^ |
| V40 Gy | 0 | 0.1% | 0.34 ^§^ |
| D2% | 14.19 Gy | 16.82 Gy | 0.041 ^§,*^ |
| **Right atrium** |  |  |  |
| D_mean_ | 5.73 Gy | 7.11 Gy | 0.087 ^+^ |
| V10 Gy | 14.6% | 21.6% | 0.29 ^§^ |
| V20 Gy | 6.7 % | 8.5 % | 0.44 ^§^ |
| V30 Gy | 2.8% | 3% | 0.38 ^§^ |
| V40 Gy | 0.09% | 0.4% | 0.17 ^§^ |
| D2% | 18.6 Gy | 24.02 Gy | 0.019 ^+,*^ |
| **Left atrium** |  |  |  |
| D_mean_ | 16.05 Gy | 17.13 Gy | 0.89 ^§^ |
| V10 Gy | 51% | 56.5% | 0.81 ^§^ |
| V20 Gy | 35% | 38.8% | 0.812 ^§^ |
| V30 Gy | 22.2% | 24% | 0.34 ^+^ |
| V40 Gy | 6 % | 5.6 % | 0.35 ^+^ |
| D2% | 37.6 Gy | 37.5 Gy | 0.64 ^§^ |
| **Left coronary** |  |  |  |
| D_mean_ | 3.14 Gy | 3.54 Gy | 0.016 ^§, *^ |
| V5 Gy | 0% | 8.45 % | 0.18 ^§^ |
| V10 Gy | 0 | 0.18% | 0.9 ^§^ |
| V20 Gy | 0 | 0.1% | 0.9 ^§^ |
| D2% | 3.55 Gy | 5.88 Gy | 0.067 ^§^ |
| **Left anterior descending** |  |  |  |
| D_mean_ | 1.8 Gy | 1.88 Gy | 0.19 ^+^ |
| D2% | 2.91 Gy | 2.94 Gy | 0.4 ^+^ |
| **Left Circumflex artery** |  |  |  |
| D_mean_ | 11.4 Gy | 13.1 Gy | 0.09 ^+^ |
| V5 Gy | 52% | 55.6% | 0.2 ^+^ |
| V10 Gy | 34% | 40% | 0.12 ^+^ |
| V20 Gy | 21% | 28% | 0.025 ^+,*^ |
| V30 Gy | 12.8% | 16.9% | 0.23 ^§^ |
| V40 Gy | 5.6% | 7.1% | 0.58 ^§^ |
| D2% | 27.55 Gy | 30.6 Gy | 0.27 ^§^ |
| **Right coronary** |  |  |  |
| D_mean_ | 1.64 Gy | 2.11 Gy | 0.057 ^+^ |
| V5 Gy | 0% | 4.4 % | 0.17 |
| D2% | 2.15 Gy | 3.03 Gy | 0.046 ^+,*^ |
| **Lung** |  |  |  |
| Volume | 5184 cm^3^ | 3913 cm^3^ | < 0.00001 ^+,*^ |
| D_mean_ | 11.63 | 12.53 | 0.0013 ^+,*^ |
| V20 Gy | 18.27% | 20% | 0.067 ^+^ |

**Supp. Table 2** Dosimetric outcomes of planning in deep inspiration breath hold (DIBH) and free breathing (FB) for the patients with middle esophageal (ME) tumors. ^+^ paired t test, ^§^ Wilcoxon signed-rank test, * : p value < 0.05
